# Effect of hydrogen peroxide on normal and acatalasemic mouse erythrocytes

**DOI:** 10.1016/j.toxrep.2020.02.001

**Published:** 2020-02-07

**Authors:** Noriyoshi Masuoka, Ayumi Zukeran, Kazunori Takemoto, Da-Hong Wang, Kohji Ishihara

**Affiliations:** a Tsudaka-Fruit Juice Laboratory, Okayama Research Park Incubation Center, 5303 Haga, Kita-ku, Okayama 701-1221, Japan; b Department of Life Science, Okayama University of Science, Okayama 700-0005, Japan; c Kake Medical Science Education Center, Okayama University of Science, Japan; d Department of Biochemistry, Okayama University of Science, Japan

**Keywords:** Acatalasemic erythrocytes, Hemoglobin oxidation, Hydrolysis-resistant erythrocytes, Membrane oxidation, Hemolysis, Takahara’s disease

## Abstract

**Objectives:**

Normal and acatalasemic mouse erythrocytes were used to clarify the relationship between oxidative damage in H_2_O_2_-treated erythrocytes and catalase activity.

**Design & Methods:**

Generation of hydrolysis-resistant erythrocytes and hemolysis were examined. The osmotic fragility test, the negative charges and the number of membrane-flickering erythrocytes among the H_2_O_2_-treated erythrocytes were investigated.

**Results:**

Small amounts of hydrolysis-resistant mouse erythrocytes were generated by treatment with 0.1 mM H_2_O_2,_ and the amount of acatalasemic erythrocytes was larger than untreated controls. Hemolysis in the acatalasemic erythrocytes was observed 30 min after the addition of the H_2_O_2_. A drastic increase in hydrolysis-resistant erythrocytes and a loss of membrane proteins in the acatalasemic erythrocytes were found as a result of the addition of 1 mM H_2_O_2_. Hemolysis in normal erythrocytes was observed at 3 mM H_2_O_2_.

**Conclusions:**

Catalase is a potent H_2_O_2_-scavenger even in acatalasemic mouse erythrocytes. It is concluded that the drastic increase of hydrolysis-resistant erythrocytes is induced by a loss of membrane function and is associated with the low catalase activity in these cells.

## Highlights

• H_2_O_2_ induces hydrolysis-resistant mouse RBCs and hemolysis.
• The hydrolysis-resistant cells were drastically increased in acatalasemic mouse RBCs.
• The increase is ameliorated by the addition of α-tocopherol.
• The increase is ascribed to a loss of membrane proteins in the RBCs.
• The increase is associated with low catalase activity in the RBCs.

## Graphical abstract

**Figure:**
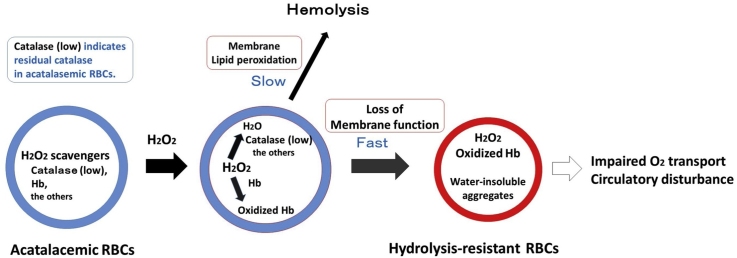

## 1 Introduction

Hydrogen peroxide (H_2_O_2_), which is one of the reactive oxygen species (ROS), induces oxidative stress in living organisms and is involved in a variety of signaling pathways [[1], [2], [3], [4]]. Antioxidants and antioxidant enzymes in blood and cells remove H_2_O_2_ and minimize the concentration to suppress the oxidative stress. The autoxidation of hemoglobin in erythrocytes is generated by H_2_O_2_ in plasma or blood removed by the erythrocytes. As the H_2_O_2_-scavenging activity of glutathione peroxidase and peroxiredoxin 2 in erythrocytes is reportedly low [5,6], it is deduced that the catalase [EC1.11.1.6] that is present in erythrocytes is important in the defense against oxidative stress. In 1948, hereditary catalase deficiency, named “acatalasemia” was reported by Takahara et al. [7,8]. People with acatalasemia suffer from progressive oral gangrene and ulceration. The disease was later called Takahara’s disease, and the authors suggested that the disease is induced by infection with H_2_O_2_–generating bacteria. During the late 1940s and early 1950s (after the war), oral hygiene in Japan was poor and the disease was present in approximately one half of the acatalasemic cases tested [[9], [10], [11]]. However, the disease is rarely found in Japan at present, with only a small number reported in China [12]. In 1961, Aebi reported cases of acatalasemia in Switzerland [13], and in 1992 Goth reported cases in Hungary [14]. The residual catalase activity of acatalasemic erythrocytes in Switzerland and Hungary was found to be higher than in Japan, and there has been no report of Takahara’s disease. The gene frequency of acatalasemia in Japan, Switzerland and Hungary is estimated to be 0.08/1000, 0.05/1000 and 0.04/1000, respectively [11]. In 2000, Goth suggested that acatalasemia patients in Hungary were at a higher risk for and an earlier manifestation of diabetes (10 years) [15]. Animal experiments indicated that low catalase activity in blood is associated with mouse diabetes under oxidative stress conditions [16]. The oxidative stress damaged oxidant-sensitive beta cells in the pancreas and thereby induced diabetes, and the beta cell damage was shown to be ameliorated by antioxidants [[17], [18], [19]].

In terms of Takahara’s disease in acatalasemic patients, it is suggested that the disease is associated with the level of residual catalase activity in erythrocytes, but the specific relationship between the disease and the residual activity level is unclear. Ogata et al. showed that methemoglobin formation is higher in acatalasemic erythrocytes than normal ones [20]. We reported that the H_2_O_2_ scavenging activity of oxidized hemoglobin is as high as the residual catalase activity in acatalasemic mouse erythrocytes [[21], [22], [23], [24]]. When supra-physiological H_2_O_2_ was added to acatalasemic mouse erythrocytes, hemolysis (0.1 mM H_2_O_2_) and hydrolysis-resistant erythrocytes (1 mM) were observed [25]. As hydrolysis-resistant erythrocytes cause impaired oxygen transport and other circulatory disturbances, we suggest that these erythrocytes are related to the disease. However, it was recently reported that normal, acatalasemic-like human erythrocytes treated with sodium azide were prepared, and hydrolysis-resistant erythrocytes were generated by a lower concentration of H_2_O_2_ than that needed for hemolysis [26,27]. We examined oxidative damage in H_2_O_2_-treated mouse erythrocytes.

## 2 Materials and methods

### 2.1 Animals and chemicals

The acatalasemic (C3H/AnLC_S_^b^C_S_^b^) and normal (C3H/AnLC_S_^a^C_S_^a^) mouse strains established by Feinstein et al. [28] were used in this study. A genetic defect has been reported [29,30], and is no report of Takahara’s disease in mice. Mice were maintained on a Laboratory diet (the CE-2 diet, Clea Japan, Tokyo, Japan) and water *ad libitum* until they were 12 weeks old. All of the animal procedures were carried out in accordance with the Guide for the Care and Use of Laboratory Animals as published by the Japanese Association for Laboratory Animal Science. All experiments were approved by the Ethics Review Committees for Animal Experimentation at Okayama University of Science. Alcian blue 8GX, 30 % H_2_O_2_ and other reagents were purchased from Wako Pure Chemical Industries (Osaka, Japan) and were of analytical grade. H_2_O_2_ solution was diluted with physiological saline containing 10 mM potassium phosphate buffer (PBS, pH 7.4), and 100 mM H_2_O_2_ (stock solution) were prepared. The concentration of H_2_O_2_ was checked using 0.01 M sodium thiosulfate solution. Catalase activity was measured according to a previously reported method [21,22]. Hemoglobin content was determined by the method of Drabkin and Austin [31], and the concentrations of hemoglobin indicated were calculated as a tetramer. Alcian-blue 8GX 400 mg was dissolved in 100 ml of PBS, and the mixture was centrifuged at 2000 X g for 10 min. The supernatant was diluted 32 times with PBS, and the solution was used as the alcian blue solution [32]. Neuraminidase (sialidase, EC 3.2.1.18) was purchased from Nakalai Tesque (Kyoto, Japan) and diluted with PBS (pH 8.0). SDS-PAGE was performed according to the method of Laemmli [33].

### 2.2 Preparation of packed erythrocytes

Mouse blood was collected by cardiac puncture, and heparin was used as an anticoagulant. Erythrocytes were separated and washed three times with PBS pH7.4 after centrifugation at 1600 X g for 10 min. Packed erythrocytes were stored at 4 °C and used immediately.

### 2.3 Hydrolysis-resistant erythrocytes among the erythrocytes treated with H_2_O_2_

The hydrolysis-resistant erythrocytes was examined as follows. Packed erythrocytes (0.06 mL) were diluted with 2.94 ml of PBS or PBS containing H_2_O_2_ (at a final concentration of 0.1, 1.0 or 5.0 mM), and the mixture was incubated at 37 °C for 5 min. The incubation mixture (0.06 mL) was diluted with 50 volumes of water. After centrifugation, the absorbance of the supernatant at 540 nm was recorded. The amount of hydrolysis-resistant erythrocytes was calculated using the absorbance (100 % hemolysis) obtained from the addition of water to the 2 % suspension. A two percent erythrocyte suspension containing 5 mM H_2_O_2_ and 50 μM α-tocopherol was prepared and tested as described above.

### 2.4 Hemolysis of mouse erythrocytes induced by H_2_O_2_

Packed erythrocytes (0.06 mL) were diluted with 2.94 ml of PBS or PBS containing H_2_O_2_, and the mixture incubated at 37 °C for 30 min. After centrifugation, the absorbance of the supernatant at 540 nm was recorded [25].

### 2.5 Osmotic fragility of H_2_O_2_-treated erythrocytes

The hemolysis of H_2_O_2_-treated erythrocytes was examined using NaCl aqueous solution [34]. Packed erythrocytes were diluted to a 2 % erythrocyte suspension (v/v) with PBS containing 0.0, 0.1, 1 or 5 mM H_2_O_2_, and the mixture was reacted at 37 °C for 5 min. Each portion (0.06 mL) was added to 2.94 ml of water or 0.40, 0.60, 0.70 and 0.90 % NaCl in water, respectively, and the mixture was incubated at 37 °C for 30 min. After centrifugation (1,600 X g for 10 min), the absorbance of the supernatant at 540 nm was recorded. Absorbance of 100 % hemolysis was obtained from the addition of water to each erythrocyte suspension treated with H_2_O_2,_ and the NaCl concentration at 50 % hemolysis was interpolated from the recorded values.

### 2.6 Preparation of 1 mM H_2_O_2_-treated erythrocytes

Packed erythrocytes (0.03 mL) were diluted with PBS containing a 1 mM H_2_O_2_ (1.47 mL) to 2 % erythrocyte ratio in suspension. The suspension was incubated at 37 °C for 5 min, and the erythrocytes were washed 3 times with PBS. After centrifugation, the packed erythrocytes were used as 1 mM H_2_O_2_-treated erythrocytes.

### 2.7 Sialic acid release from mouse erythrocytes by sialidase treatment

As the negative charge of erythrocytes is associated with sialic acid content, the sialic acid released by sialidase was measured [35].

Packed erythrocytes or 1 mM H_2_O_2_-treated erythrocytes (0.03 mL) were diluted with 1.47 ml of PBS containing sialidase (1 unit/ mL). Each suspension was incubated at 37 °C for 30 min. The supernatant was separated by centrifugation and was stored at -80 °C until measurement. The sialic acid determination was carried out using a spectrophotometric assay kit (BioVision Inc., USA) according to the manufacturer’s suggested procedure, and the absorbance at 570 nm was recorded. The packed erythrocytes were used as sialidase-treated erythrocytes for the following experiment.

### 2.8 Effect of 1 mM H_2_O_2_ on alcian blue binding to erythrocytes and sialidase-treated erythrocytes

The negative charge on the erythrocytes was evaluated using the alcian blue binding method.

The packed erythrocytes or sialidase-treated erythrocytes were diluted with PBS (1.47 mL) or PBS containing 1 mM H_2_O_2_, and the mixture was reacted at 37 °C for 5 min. After centrifugation, the erythrocytes were diluted with PBS to 1.0 × 10^5^ erythrocytes / μL. Nine volumes of alcian blue solution were added to each suspension. The mixture was reacted at 37 °C for 30 min. After centrifugation (800 X g for 10 min), absorbance of the supernatant at 650 nm was recorded to determine the unbound alcian blue. Evaluation of the negative charge was carried out using a reported method [36].

### 2.9 Membrane-flickering erythrocytes after H_2_O_2_ treatment

The vibrating erythrocytes were counted under microscopy [37].

Packed erythrocytes (0.03 mL) or 1 mM H_2_O_2_-treated erythrocytes were diluted with 400 volumes of PBS, and all of the erythrocytes as well as the flickering ones were counted with a Thoma blood counter. The percentage of the flickering erythrocytes was calculated as (vibrating erythrocytes / total erythrocytes) X 100.

### 2.10 SDS-PAGE Analysis of membrane proteins in H_2_O_2_-treated erythrocytes

As SDS-PAGE of H_2_O_2_-treated mouse erythrocytes has been reported in [25], SDS-PAGE of membrane proteins in the erythrocytes were examined.

Three ml of PBS or 0.1, 0.5, 1.0 and 5.0 mM H_2_O_2_ in PBS were added to packed erythrocytes (0.06 mL). The mixture was incubated at 37 °C for 5 min. The mixture was centrifuged at 1,600 X g for 10 min. Three ml of water were added to the packed erythrocytes, and the mixture was centrifuged at 11,000 X g for 15 min. A sample buffer containing 2-mercaptoethanol was added to the residue. The samples were applied to a 7 % polyacrylamide gel and electrophoresis was carried out. The proteins in the gels were stained with Coomassie Brilliant Blue R-250.

The gel was blotted on a nitrocellulose membrane. Band 3 was stained using the band 3 antibody (ab104998, Abcam Co., UK), goat antibody to rabbit IgG (HRP activity) (ab97051) and 3, 3′, 5, 5′-tetramethylbenzidine-H_2_O_2_ solution (Ez West Blue, Atto Co., Tokyo, Japan). Oxidized proteins were stained using a protein-carbonyls detection kit (Cosmo Bio Co. Ltd., Tokyo) [38], goat antibody to rabbit IgG (111-036-144 Jackson immune Res. Lab., Inc., PA, USA) and Ez West Blue.

### 2.11 Statistical analyses

Data were the mean ± SE and analyzed using Student's *t*-test. A difference of p < 0.05 was considered significant.

## 3 Results

Mouse catalase activity was examined in the presence of 70 μM H_2_O_2_ at 25 °C, since the activity of the mutant catalase was deactivated at 37 °C [22,23]. The activity in acatalasemic and normal erythrocytes at 25 °C was 1.05 ± 0.07 and 7.27 ± 0.63 μmol/s/g of hemoglobin, respectively. A two percent mouse erythrocyte suspension contained 64.5 ± 9.9 μM hemoglobin.

### 3.1 Percentage of hydrolysis-resistant mouse erythrocytes

The hydrolysis-resistant erythrocytes treated with 0–5 mM H_2_O_2_ were examined. Spontaneous hydrolysis-resistant erythrocytes in normal and acatalasemic erythrocytes were hardly detected. When erythrocytes were treated at 0.1 mM H_2_O_2_, a small percentage of hydrolysis-resistant erythrocytes was observed **(**Table 1**)**. The amount of the resistant acatalasemic erythrocytes was higher than that in normal erythrocytes. When erythrocytes were treated at 1 mM H_2_O_2_, the hydrolysis-resistant acatalasemic erythrocytes dramatically increased to 90.1 %, but the ratio in the normal erythrocytes was 15.0 %. The effect in the hydrolysis-resistant acatalasemic erythrocytes induced by 5 mM H_2_O_2_ was ameliorated by the addition of 50 μM α-tocopherol. This suggests that the drastic increase in the hydrolysis-resistant erythrocytes is associated with changes in membrane function.

**Table 1:** Hydrolysis-resistant erythrocytes (%) in H_2_O_2_-treated mouse erythrocytes.

H_2_O_2_ (mM)	Vitamin E	Normal mice (n≥4)	Acatalasemic mice (n≥4)
0.0	–	1.5 ± 0.9	1.0 ± 0.5
0.1	–	3.3 ± 3.0	11.5 ± 2.7
1.0	–	15.0 ± 2.5	90.1 ± 6.2*
5.0	–	14.0 ± 4.5	94.3 ± 10.0*
5.0	+	ND	19.0 ± 2.7**

ND is “not determined”. *p < 0.01 compared to the value in normal mice. **p < 0.01 compared to the value in the absence of vitamin E.

### 3.2 Hemolysis of mouse erythrocytes induced by H_2_O_2_

Acatalasemic mouse erythrocytes slowly exhibited hemolysis (85 ± 12 %) in the presence of 0.1 mM H_2_O_2_, and normal mouse erythrocytes did the same in 3 mM H_2_O_2_ (80 ± 5 %). This indicates that concentration of H_2_O_2_, which causes hemolysis, is associated with catalase activity.

### 3.3 Osmotic fragility of H_2_O_2_-treated erythrocytes

The osmotic fragility of H_2_O_2_-treated erythrocytes was examined to study whether the hemolysis was associated with the membrane proteins of the erythrocytes. The concentration of NaCl at 50 % hemolysis of normal erythrocytes was the same as that of acatalasemic erythrocytes in the absence of H_2_O_2_ **(**Table 2**)**. Hypotonic hemolysis of 0.1 mM H_2_O_2_-treated acatalasemic erythrocytes was observed, but in normal erythrocytes it was observed at 5 mM H_2_O_2_. The hemolysis of the acatalasemic erythrocytes at 0.1 mM H_2_O_2_ was ameliorated by the addition of 50 μM α-tocopherol. These results suggest that hemolysis is induced by membrane fluidity changes in the erythrocytes.

**Table 2:** Sodium chloride concentration (%) at 50 % hemolysis.

H_2_O_2_ (mM)	Vitamin E	Normal mice (n≥5)	Acatalasemic mice (n≥5)
0.0	–	0.65 ± 0.02	0.65 ± 0.01
0.1	–	0.67 ± 0.02	0.72 ± 0.01*
0.1	＋	0.66 ± 0.02	0.65 ± 0.02
1.0	–	0.67 ± 0.02	ND
5.0	–	0.71 ± 0.02*	ND

ND is “not determined”. *p < 0.01 compared to the value in the absence of H_2_O_2_.

### 3.4 Sialic acid release from erythrocytes treated with sialidase

The sialic acid released by the administration of sialidase from 2 % acatalasemic and normal mouse erythrocytes (n = 7) was low, being 0.02 ± 0.02 and 0.01 ± 0.01 mmol / L, respectively. Upon the administration of 1 mM H_2_O_2_, the sialic acid released by sialidase from the acatalasemic erythrocytes was 0.08 ± 0.02 mmol / L, which is significantly higher than from the normal erythrocytes (0.04 ± 0.01 mmol / L). This suggests that 1 mM H_2_O_2_ induced greater damage in the membrane on acatalasemic erythrocytes than normal erythrocytes. However, as sialic acid residues on erythrocyte membrane are oxidized or depolymerized by H_2_O_2_ [39], we examined the negative charge of these erythrocytes as follows.

### 3.5 Alcian blue binding to erythrocytes and sialidase-treated erythrocytes

**Fig. 1:**
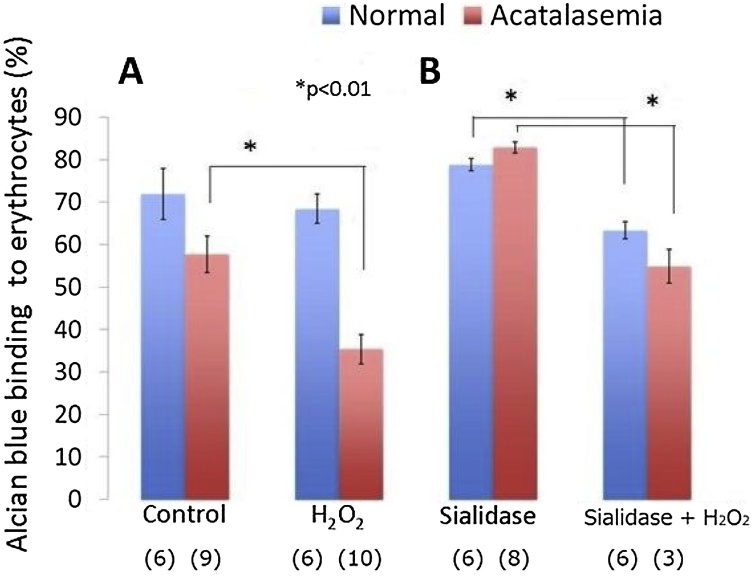
Alcian blue binding to erythrocytes. (A) Control: erythrocytes were treated with PBS. H_2_O_2_: erythrocytes were treated with 1 mM H_2_O_2_. (B) Sialidase: erythrocytes were treated with sialidase; Sialidase + H_2_O_2_: sialidase-treated erythrocytes were treated with 1 mM H_2_O_2_. The binding to erythrocytes was monitored by the absorbance at 650 nm. The binding values were calculated from the absorbance compared to control alcian blue solution (100 %, A_650 nm_ = 0.067). The numbers in parentheses indicate the number of mice used.

As the binding to erythrocytes of the cationic pigment alcian blue is proportional to the negative charge on the erythrocytes, the effect of 1 mM H_2_O_2_ on the binding of the alcian blue to erythrocytes and sialidase-treated erythrocytes was examined. The binding to normal erythrocytes was almost the same as the binding to acatalasemic erythrocytes in the absence of H_2_O_2_ (Fig. 1A). However, the pigment binding to 1 mM H_2_O_2_-treated acatalasemic mouse erythrocytes significantly decreased (23 %) compared to that (4 %) in normal erythrocytes. Alcian blue binding to sialidase-treated erythrocytes is shown in Fig. 1B. When the sialidase-treated erythrocytes were reacted with 1 mM H_2_O_2_, the binding to both types of mouse erythrocytes significantly decreased. The decrease in binding to acatalasemic mouse erythrocytes (30 %) was larger than that in normal erythrocytes (16 %).

These results confirmed that the addition of 1 mM H_2_O_2_ induces potent damage on the membrane of acatalasemic erythrocytes.

### 3.6 Numbers of flickering erythrocytes after H_2_O_2_ treatment

**Fig. 2:**
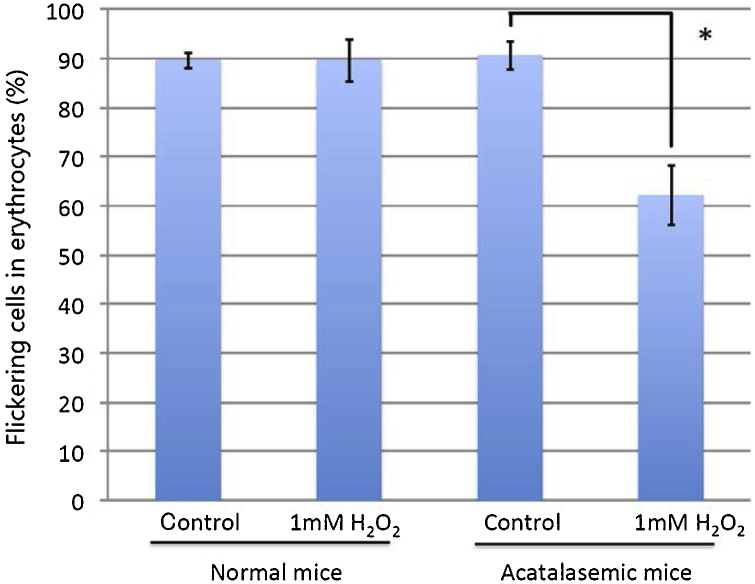
Percentages of flickering erythrocytes in the presence of 1 mM H_2_O_2_. * indicates P < 0.01.

Erythrocyte membrane flickering is maintained by membrane proteins and an ATP mechanism [40,41]. When acatalasemic erythrocytes were treated with 1 mM H_2_O_2_, the flickering significantly decreased (62.2 ± 6.2 %) compared to the control erythrocytes (Fig. 2). However, in normal erythrocytes, there was no significant difference from the control erythrocytes. The decrease in acatalasemic erythrocytes may be due to suicidal erythrocyte death, eryptosis, induced by H_2_O_2_ [42].This suggests that 1 mM H_2_O_2_ induces oxidation of hemoglobin, as well as the inactivation of glycolytic enzymes and other, non-specific reactions in acatalasemic erythrocytes.

### 3.7 SDS-PAGE Analysis of membrane proteins in H_2_O_2_-treated erythrocytes

**Fig. 3:**
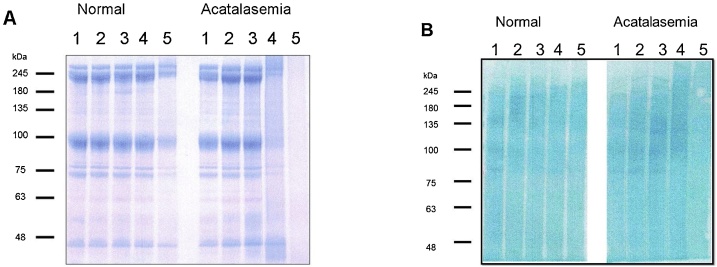
SDS-PAGE of mouse membrane-proteins from erythrocytes treated with various concentrations of H_2_O_2_.

The SDS-PAGE of line 1 (control) indicates the membrane proteins, band 1 (spectrin-α, 240 kDa), band 2 (spectrin-β, 220 kDa), band 2.1 (ankyrin, 210 kDa), band 3 (anion exchanger, 95 kDa), band 4.1 (80 kDa), band 4.2 (72 kDa) and band 5 (actin, 43 kDa) of mouse erythrocytes **(**Fig. 3A**)**. The band 3 protein was confirmed using an antibody. In acatalasemic erythrocytes, 0.1-0.5 mM H_2_O_2_ did not affect the size or amount of the membrane proteins (Fig. 3A right, line 2, 3). The membrane proteins (broad bands of 80–250 kDa) were faintly stained with a protein-carbonyl immunohistochemical stain (Fig. 3B right line 2, 3). In 1 mM H_2_O_2_-treated acatalasemic erythrocytes (Fig. 3A right line 4), the membrane proteins mostly disappeared and a new band of high molecular-weight aggregates (>250 kDa) appeared. This suggests that the membrane proteins were converted to water-insoluble aggregates by the H_2_O_2_. In contrast, the pattern of normal erythrocytes was not affected by 0.1–1.0 mM H_2_O_2_, and a decrease in membrane-proteins was observed only at 5 mM H_2_O_2_ (Fig. 3A left, line 1-5).

(A) Coomassie Brilliant Blue R-250 stain, (B) protein-carbonyl immunohistochemical stain. The left edge of the gel provides the molecular size markers. Line 1 is from erythrocytes, line 2 is from 0.1 mM H_2_O_2_ treated-erythrocytes, line 3 is from 0.5 mM H_2_O_2_, line 4 is from 1.0 mM H_2_O_2_, line 5 is from 5.0 mM H_2_O_2_.

## 4 Discussion

As erythrocytes are oxygen carriers, autoxidation of hemoglobin generates endogenous H_2_O_2_ in these cells, and the concentration of physiological H_2_O_2_ in plasma is reported to be 1–5 μM [2]. The residual catalase and hemoglobin in acatalasemic mouse erythrocytes are potent H_2_O_2_-scavengers in erythrocytes. Hydrolysis-resistant erythrocytes were hardly observed under physiological conditions, although a faint ESR signal (g = 2.005) of ferryl hemoglobin radicals, oxidized hemoglobin, was detected in the acatalasemic erythrocytes [25]. This suggests that endogenous H_2_O_2_ and the radicals were removed by cytosolic catalase and other scavenging activities in the erythrocytes. Small amounts of hydrolysis-resistant erythrocytes were detected after the addition of 0.1 mM (supra-physiological) H_2_O_2_, and the amount in the acatalasemic erythrocytes was larger than in the normal ones **(**Table 1**)**. This may be explained by the fact that 0.1 mM H_2_O_2_ oxidizes cytosolic hemoglobin and generates hydrolysis-resistant erythrocytes, a process which is associated with the membrane rigidity induced by H_2_O_2_ *via* formation of membrane bound hemoglobin [26,27]. Approximately 30 min after 0.1 mM H_2_O_2_ addition, hemolysis was observed in most of the acatalasemic erythrocytes, and this effect was suppressed by the addition of α-tocopherol. The 0.1 mM H_2_O_2_-treated acatalasemic erythrocytes exhibited osmotic fragility **(**Table 2**)**. From these results, it is concluded that hemolysis was induced by membrane lipid peroxidation.

However, when the concentration of exogenous H_2_O_2_ was increased to 1 mM, the hydrolysis-resistant erythrocytes were drastically increased in the acatalasemic erythrocytes. The drastic increase was prevented by α–tocopherol, and a decrease in the water-soluble membrane proteins on SDS-PAGE was observed (Fig. 3).These results show that 1 mM H_2_O_2_ induces a loss of membrane structure and function. The decrease of alcian blue binding to erythrocytes along with the membrane-flickering erythrocytes indicated membrane property change and disturbed metabolism, and suggested eryptosis in the erythrocytes [42,43]. From these results, 1 mM H_2_O_2_ freely reacts with cytosolic hemoglobin to generate a high concentration of ferryl hemoglobin radicals and the aggregation reactions in turn cause the formation of hydrolysis-resistant erythrocytes. The formation of high molecular-weight aggregates in association with these changes may involve in ischemic stroke [44].

As the drastic increase of hydrolysis-resistant erythrocytes induced by supra-physiological H_2_O_2_ was associated with low catalase activity in erythrocytes, we compared the residual catalase activity in mouse acatalasemic erythrocytes with that in Japanese acatalasemic cases. Based on the published report [45], the residual catalase activity of Japanese acatalasemic erythrocytes was 0.13 % of the normal activity (at 37 °C), while the mouse residual activity at 25 °C is estimated to be 2.6 % of the human catalase activity [22,23]. As the mouse residual catalase activity is twenty-times higher than the human activity, we suggest that the mouse residual catalase in erythrocytes has a sufficient capacity to scavenge the H_2_O_2_ induced by the infection of H_2_O_2_-generating bacteria, which will develop the onset of Takahara’s disease, but the human residual catalase activity does not [8]. It may be important to recommend that acatalasemic persons take α-tocopherol since it ameliorates several oxidative stresses induced by H_2_O_2_ or alloxan administration under acatalasemic conditions [[17], [18], [19]].

## Author contributions statement

N. Masuoka designed the experiments and wrote the manuscript. All authors discussed and approved the manuscript.

## Funding statement

The authors received no funding from an external source.

## Additional information

No additional information is available for this paper.

## Declaration of Competing Interest

The authors declare no conflict of interest.

## References

[1] Halliwell B., Clement M.V., Long L.H. (2000). Hydrogen peroxide in the human body. FEBS Lett..

[2] Forman H.J., Bernardo A., Davies K.J.A. (2016). What is the concentration of hydrogen peroxide in blood and plasma?. Arch. Biochemi. Biophys..

[3] Veskoukis A., Kerasioti E., Oriftis A., Kouka P., Spanidis Y., Makri S., Kouretas D. (2019). A battery of translational biomarkers for the assessment of the *in vitro* and *in vivo* antioxidant action of plant polyphenolic compounds: the biomarker issue. Curr. Opin. Toxicol..

[4] Veskoukis A.S., Paschalis V., Kypatos A., Nikolaidis M.G. (2018). Administration of exerce-conditioned plasma alters muscle catalase kinetics in rat: an argument for *in vivo*-like Km instead of *in vitro*-like Vmax. Redox Biol..

[5] Nagababu E., Chrest F.J., Rifkind J.M. (2003). Hydrogen-peroxide-induced heme degradation in red blood cells: the protective roles of catalase and glutathione peroxidase. Biochim. Biophys. Acta.

[6] Low F.M., Hampton M.B., Peskin A.V., Winterbourn C.C. (2007). Peroxiredoxin 2 functions as a noncatalytic scavenger of low-level hydrogen peroxide in the erythrocyte. Blood.

[7] Takahara S., Miyamoto H. (1948). Three cases of progressive oral gangrene due to lack of catalase in the blood. Jpn J. Otol..

[8] Takahara S. (1952). Progressive oral gangrene probably due to lack of catalase in the blood (acatalasemia); report of nine cases. Lancet.

[9] Ogata M. (1991). Acatalasemia. Human Genet..

[10] Eaton J.W., Ma M., Scriver C.R., Beaudet A.L., Sly W.S., Valle D. (1995). Acatalasemia. The Metabolic and Molecular Bases of Inherited Disease.

[11] Goth L., Nagy T. (2013). Inherited catalase deficiency: is it benign or a factor in various age related disorders?. Mutat. Res..

[12] Wang Q., Ni J., Zhang X., Li Y., Xuan D., Zhang J. (2014). Long-term follow-up evaluation of an acatalasemia boy with severe periodontitis. Clin. Chim. Acta.

[13] Aebi H., Heiniger J.P., Buetler R., Haessiga A. (1961). Two cases of acatalasemia in Switzerland. Experentia.

[14] Góth L. (1992). Two cases of acatalasemia in Hungary. Clin. Chim. Acta.

[15] Góth L., Eaton J.W. (2000). Hereditary catalase deficiencies and increased risk of diabetes. Lancet.

[16] Takemoto K., Tanaka M., Iwata H., Nishihara R., Ishihara K., Wang D.H., Ogino K., Taniuchi K., Masuoka N. (2009). Low catalase activity in blood is associated with the diabetes caused by alloxan. Clin. Chim. Acta.

[17] Kikumoto Y., Sugiyama H., Inoue T., Morinaga H., Takiue K., Kitagawa M., Fukuoka N., Saeki M., Maeshima Y., Wang D.H., Ogino K., Masuoka N., Makino H. (2010). Sensitization to alloxan-induced diabetes and pancreatic cell apoptosis in acatalasemic mice. Biochim. Biophys. Acta.

[18] Kamimura W., Doi W., Takemoto K., Ishihara K., Wang D.H., Sugiyama H., Oda S., Masuoka N. (2013). Effect of vitamin E on alloxan-induced mouse diabetes. Clin. Biochem..

[19] Takemoto K., Doi W., Masuoka N. (2016). Protective effect of vitamin E against alloxan-induced mouse hyperglycemia, Biochim. Biophys. Acta.

[20] Ogata M., Kobayashi H., Ioku N., Ishii K. (1986). Methemoglobin concentration in the blood of acatalasemic mice. Proc. Jap. Acad..

[21] Masuoka N., Wakimoto M., Ubuka T., Nakano T. (1996). Spectrophotometric determination of hydrogen peroxide: catalase activity and rates of hydrogen peroxide removal by erythrocytes. Clin. Chim. Acta.

[22] Masuoka N., Wakimoto M., Ohta J., Ishii K., Nakano T. (1997). Characterization of hydrogen peroxide removal activities in mouse hemolysates: catalase activity and hydrogen peroxide removal activity by hemoglobin. Biochim. Biophys. Acta.

[23] Wakimoto M., Masuoka N., Nakano T., Ubuka T. (1998). Determination of glutathione peroxidase activity and its contribution to hydrogen peroxide removal in erythrocytes. Acta Med.Okayama.

[24] Masuoka N., Kodama H., Abe T., Wang D.H., Nakano T. (1637). Characterization of hydrogen peroxide removal reaction by hemoglobin in the presence of reduced pyridine nucleotides. Biochim. Biophys. Acta.

[25] Masuoka N., Sugiyama H., Ishibashi N., Wang D.H., Masuoka T., Kodama H., Nakano T. (2006). Characterization of acatalasemic erythrocytes treated with low and high dose hydrogen peroxide: hemolysis and aggregation. J. Biol. Chem..

[26] Nascimento H., Belo L., Fernandes J., Rocha S., Quintanilha A., Santos-Silva A. (2010). In vitro studies with’ acatalasemic-like’ erythrocytes and hydrogen peroxide: attention to the formation of lysis resistant erythrocytes. Int. J. Lab. Hematol..

[27] Mendanha S.A., Anjos J.L.V., Silva A.H.M., Alsoso A. (2012). Electron paramagnetic resonance study of lipid and protein membrane components of erythrocytes oxidized with hydrogen peroxide. Braz. J. Med. Biol. Res..

[28] Feinstein R.N., Braun J.T., Howard J.B. (1967). Acatalasemic and hypocatalasemic mouse mutants. Mutational variations in blood and solid tissue catalases. Arch. Biochem. Biophys..

[29] Shuffer J.B., Preston K.E. (1990). Molecular analysis of an acatalasemic mouse mutant. Biochem. Biophys. Res. Commun..

[30] Wang D.H., Tsutsui K., Sano K., Masuoka N., Kira S. (2001). cDNA cloning and expression of mutant catalase from hypocatalasemic mouse: comparison with the acatalasemic mutant. Biochim. Biophys. Acta.

[31] Drabkin D.L., Austin J.H. (1935). Spectrophotometric studies: II. Preparations from washed blood cells; nitric oxide hemoglobin and sulfhemoglobin. J. Biol. Chem..

[32] Levin M., Smith C., Walters M.D., Gascoine P., Baratt T.M. (1985). Steroid-responsive nephrotic syndrome: a generalised disorder of membrane negative charge. Lancet.

[33] Laemmli U.K. (1970). Cleavage of structural proteins during the assembly of the head of bacteriophage T4. Nature.

[34] Dumont A.E., Nachbar M.S., Martelli A.B. (1977). Altered erythrocyte osmotic fragility in mice with Ehrlich ascites tumor. J. Natl. Cancer Inst..

[35] Eylar E.H., Madoff M.A., Brody O.V., Oncley J.L. (1962). The contribution of sialic acid to the surface charge of the erythrocyte. J. Biol. Chem..

[36] Bernard A.M., Amor A.O., Lauwerys R.R. (1988). Decrease of erythrocyte and glomerular membrane negative charges in chronic cadmium poisoning. Br. J. Ind. Med..

[37] Blowers R., Clarkson E.M., Maizels M. (1951). Flicker phenomenon in human erythrocytes. J. Physiol. (Paris).

[38] Nakamura A., Goto S. (1996). Analysis of protein carbonyls with 2,4-dinitrophenyl hydrazine and its antibodies by immunoblot in two-dimensional gel electrophoresis. J. Biochem..

[39] Neyra C., Paladino J., Le Borgne M. (2015). Mechanisms of depolymerization and activation of a polysialic acid and its tetramer by hydrogen peroxide. Carbohydr. Polym..

[40] Betz T., Lenz M., Joanny J.-F., Sykes C. (2009). ATP-dependent mechanics of red blood cells. Proc. Natl. Acad. Sci..

[41] Puckeridge M., Chapman B.E., Conigrave A.D., Kuchel P.W. (2014). Membrane flickering of the human erythrocyte: physical and chemical effectors. Eur. Biophys. J..

[42] Briglia X.M., Fazio A., Faggio C., Lang F. (2015). Triggering of suicidal erythrocyte death by zosuquidar. Cell. Physiol. Biochem..

[43] Briglia Y.M., Rossi M.A., Faggio C. (2017). Eryptosis: Ally or Enemy. Curr. Med. Chem..

[44] Santos-Silva A., Robelo I., Castro E., Belo L., Catarino C., Monteiro I., Almeida M.D., Quintanilha A. (2002). Erythrocyte damage and leukocyte activation in ischemic stroke. Clin. Chim. Acta.

[45] Ogata M., Mizugaki J., Taketa K., Takahara S. (1977). Activities of catalase in leucocytes and glucose-6-phosphate dehydrogenase in erythrocytes of hypocatalasemia and acatalasemia. Tohoku J. Exp. Med..

